# HtrA of Borrelia burgdorferi Leads to Decreased Swarm Motility and Decreased Production of Pyruvate

**DOI:** 10.1128/mBio.01136-18

**Published:** 2018-07-10

**Authors:** James L. Coleman, Alvaro Toledo, Jorge L. Benach

**Affiliations:** a Center for Infectious Diseases, Stony Brook University, Stony Brook, New York, USA; Albert Einstein College of Medicine

**Keywords:** *Borrelia*, glycolytic enzymes, HtrA, P66, swarm motility

## Abstract

Borrelia burgdorferi HtrA (HtrABb) is a serine protease that targets damaged or improperly folded proteins. In our previous studies, HtrABb specifically degraded basic membrane protein BmpD, chemotaxis phosphatase CheX, and outer membrane protein P66. In addition, HtrABb degrades virulence factor BB0323 and components of the extracellular matrix fibronectin and aggrecan. A proteomics-based analysis (two-dimensional difference gel electrophoresis [2-D DIGE], liquid chromatography-mass spectrometry [LC-MS]) of an HtrABb-overexpressing strain of B. burgdorferi (A3HtrAOE) revealed that protein levels of P66 were reduced in comparison to wild-type B. burgdorferi, confirming its status as an HtrABb substrate. Hbb, a P66-DNA-binding transcription factor, was specifically degraded by HtrABb, providing supportive evidence for a role for both in the regulation of P66. A3HtrAOE exhibited reduced motility in swarm assays, a possible link between overabundance of HtrABb and its enzymatic specificity for P66. However, the ΔP66 strain did not have reduced motility in the swarm assays, negating a role for this protein. The proteomics analyses also identified three enzymes of the glycolytic pathway, glyceraldehyde-3-phosphate dehydrogenase (GAPDH), glycerol-3-phosphate dehydrogenase (GPDH), and glycerol kinase (GK), and one enzyme involved in carbohydrate metabolism, diphosphate-fructose-6-phosphate 1-phosphotransferase, which were reduced in A3HtrAOE. Consistent with its reduced protein levels of these glycolytic enzymes, A3HtrAOE was also deficient in production of pyruvate. We propose a model for a role for HtrABb in contributing to a decrease in metabolic activity of B. burgdorferi.

## IMPORTANCE

Being a vector-borne bacterium, B. burgdorferi must remodel its protein content as it transfers from tick to mammal. Proteolysis is a mechanism whereby remodeling can be accomplished. HtrABb degrades a number of proteins whose disappearance may help in preparing this organism for a stage of low metabolic activity.

## INTRODUCTION

Borrelia burgdorferi HtrA (HtrABb, BB0104) is an immunogenic protease whose fundamental structural unit is a trimer and has the catalytically active proteolytic domain, the Ser-His-Asp catalytic triad (1). HtrABb induces the formation of antibodies in laboratory mice as well as in patients. Although immunization of mice with recombinant HtrABb elicited strong antibody responses (1), these did not lead to protection (1, 2).

HtrABb has been studied by several laboratories, and it is clear that this protease has multiple and diverse proteolytic and chaperone functions (3). HtrABb selectively degrades several endogenous proteins. The first identified substrate was virulence factor BB0323, a C-terminal LysM-like domain-containing protein (4) that is processed by HtrABb into two N- and C-terminal peptides (5). We identified basic membrane protein D (BmpD/BB0385), chemotaxis phosphatase CheX (BB0671), and outer membrane integral protein P66 as HtrABb substrates (1). HtrABb is inhibited by zinc, a trait that is shared by other proteases of this family (6).

HtrABb promotes invasiveness through degradation of the extracellular matrix components aggrecan (7) and fibronectin (8), an unexpected role for a periplasmic protease. HtrABb is also released in vesicles (9). However, this exogenous role is not unique to the HtrA of B. burgdorferi. Overexpression of HtrA in Helicobacter pylori also leads to increased HtrA secretion and to cleavage of E-cadherin. This function increases bacterial transmigration and delivery of the type IV secretion system (T4SS) effector protein CagA into polarized epithelial cells (10). H. pylori secretes HtrA through a T4SS to deliver CagA to cells. In this pathway, HtrA degrades epithelial junctional proteins necessary for this bacterium to reach the specific cell basolateral locations where CagA is injected (11). Thus, it is evident that the HtrAs of these two pathogens have important exogenous proteolytic roles that result in enhanced invasiveness.

Our laboratory has reported that cholesterol glycolipids aggregate in the form of lipid raft microdomains in both the outer (12) and inner (13) membranes of B. burgdorferi. These lipid rafts associate with a diverse set of proteins (12, 14), which are involved in the formation and maintenance of these microdomains. HtrABb partitions consistently in lipid raft domains from the outer and inner membranes of B. burgdorferi, suggesting that it is present in several subcellular compartments (13, 15), so it is not surprising that it can be exported. H. pylori incorporates cholesterol into its membrane(s) as well (16), and HtrA is surface exposed on Ehrlichia chaffeensis and has the ability to degrade endogenous surface-exposed proteins (17, 18), which may be an important common feature of these pathogens for exporting or releasing HtrA into the environment. It is becoming rapidly apparent that HtrAs from a diverse group of bacterial pathogens are not just confined to the periplasm but are secreted into the environment, and this includes the HtrA of B. burgdorferi. The secretion of HtrA when used for invasion or for acquisition of nutrients by an increasing number of bacterial pathogens could represent a new strategy for disease pathogenesis (19).

To investigate the role of HtrABb in protein levels *in vivo*, we looked for changes in the B. burgdorferi protein profile by two-dimensional (2-D) gel electrophoresis analysis using a strain engineered to overexpress HtrABb (A3HtrAOE) (20). We identified the outer membrane integral protein P66 as a proteolytic target for HtrABb, both *in vitro* and *in vivo*. HtrABb and P66 partition into the detergent-resistant membrane fraction (DRM) when treated with Triton X-100, which strongly suggests proximity within the membranes. Membrane colocalization and the likelihood of protein-protein interaction point to a potential regulatory role for HtrABb with respect to P66 expression. In addition to the proteolysis of P66, the overexpression of HtrABb was shown to have an inhibitory effect on *p66* transcript level in A3HtrAOE, suggesting multilevel regulation.

P66 is a well-studied, surface-exposed, integral outer membrane protein that is a ligand for β3-chain integrins (21–23). P66 also functions as a porin (24–28). P66 is not expressed while spirochetes are in the midgut of the unfed tick. Upon feeding with blood, P66 begins to be expressed, with production lasting until the blood meal has been digested (29). Moreover, P66 is required for the vascular transmigration of this organism (30). Studies using differential culture conditions suggested that P66 production is regulated at the transcriptional as well as posttranscriptional level (29). Our studies reached the same conclusion (20). Hbb is the homolog of DNA-binding proteins such as hydroxyurea (HU)-like proteins and integration host factor (IHF) or histone-like proteins (31). Recombinant Hbb bound the *p66* promoter region with high affinity and specificity in electrophoretic mobility shift assays, suggesting a transcriptional role for this molecule (32). Thus, the effect of HtrABb on Hbb was also of interest.

In this study, we sought to expand on the regulation of P66 by HtrA and to identify additional substrates. A strong reduced-motility phenotype in the overexpresser A3HtrAOE led us to the formulation of a model for the roles of HtrABb in the physiology of B. burgdorferi.

## RESULTS

### 2-D difference gel electrophoresis (2-D DIGE) reveals decreased levels of P66 and three glycolytic enzymes in the HtrABb-overexpressing strain A3HtrAOE.

Outer membrane protein P66 and chemotaxis phosphatase CheX were previously identified as the substrates for HtrABb using biochemical and genetic approaches. These approaches included (i) immunoprecipitation (pulldown) assays using rabbit anti-HtrABb to discover binding partners in wild-type (WT) B31A3, (ii) Coomassie blue-stained 2-D gel electrophoresis of wild-type B31A3 and HtrA-overexpressing A3HtrAOE strains, and (iii) *in vitro* digestion of recombinant proteins (1, 20).

**FIG 1:**
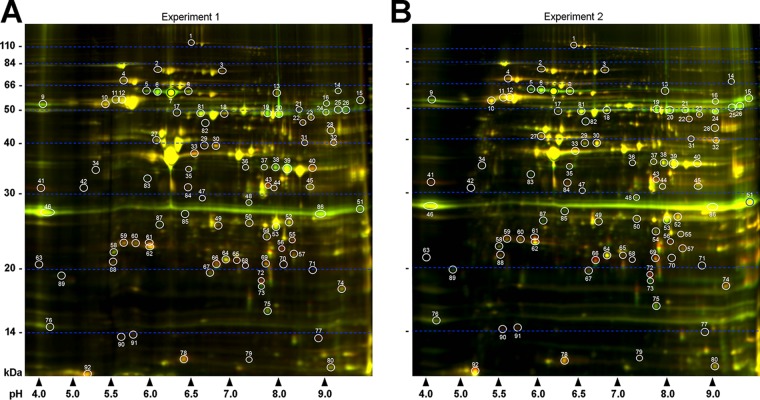
Analysis of wild-type B. burgdorferi B31A3 and HtrA-overexpressing strain A3HtrAOE by two-dimensional difference gel electrophoresis (2-D DIGE) revealed numerous differentially expressed proteins. Depicted are two independent experiments in which lysates of mid-log-phase B31A3 (green) and A3HtrAOE (red) were coanalyzed by isoelectric focusing (pH 3 to 10) followed by 12% SDS-PAGE. (A) In experiment 1, a total of 92 differentially expressed protein spots were detected (white circles and numbers). (B) In experiment 2, the same 92 protein spots were detected. Molecular weight standards are indicated on the left of each panel, and pH values are indicated on the bottom.

To expand these findings and gain new insight into the role of HtrABb and to identify additional substrates, we used 2-D DIGE to further analyze changes in protein levels in A3HtrAOE compared to wild-type B31A3. In two independent, identical experiments, lysates from equal numbers (5 × 10^9^) of B31A3 and A3HtrAOE cells, grown at 33°C, were stained with different-color fluor dyes (B31A3, green; A3HtrAOE, red). The samples were subsequently mixed and separated by isoelectric focusing (IEF) on one immobilized pH gradient (IPG) strip (pH 3 to 10), followed by 12% SDS-PAGE. Following acquisition of 2-D DIGE images, 92 protein spots were selected and quantitated over the course of two independent experiments by the DeCyder 2-D differential analysis software. This software increases throughput by measuring changes in protein spots with built-in Student’s *t* test and analysis of variance (ANOVA) providing statistical accuracy. The 92 protein spots were chosen for their likelihood to be identified by mass spectroscopy. Of these, 64 protein spots (red font) in A3HtrAOE had either increased or decreased (denoted by minus sign) levels in comparison to B31A3 in one or both experiments (see Table S1 in the supplemental material). Of this total, 44 were different in both experiments. Of these, 22 had higher protein levels and 22 were reduced in A3HtrAOE in comparison to B31A3. A total of 28 spots were unaffected in either experiment (Fig. 1; Table S1).

10.1128/mBio.01136-18.1 TABLE S1  Identification of selected proteins from 2-D DIGE of B. burgdorferi 698 (pH 3 to 10). Download TABLE S1, DOCX file, 0.1 MB. Copyright © 2018 Coleman et al. 2018 Coleman et al. This content is distributed under the terms of the Creative Commons Attribution 4.0 International license.

**FIG 2:**
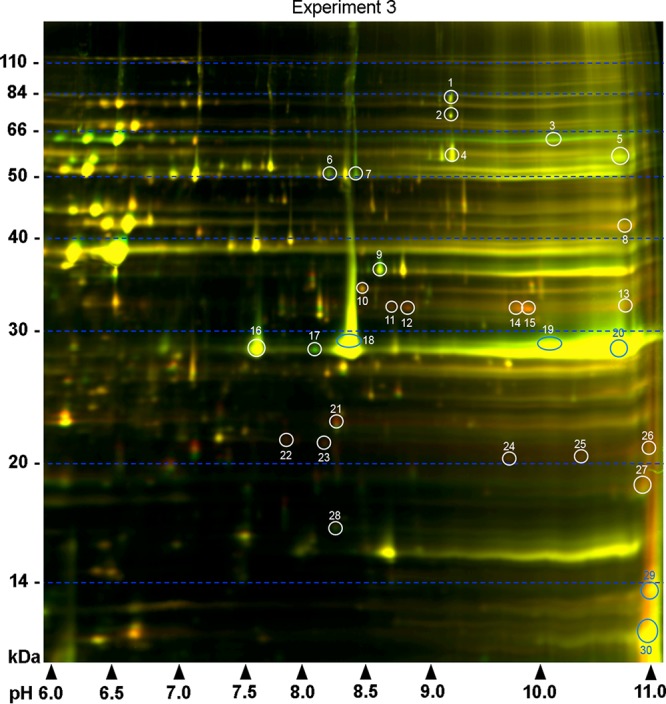
Further analysis of the basic proteins on the right side of the gels from experiments 1 and 2 (Fig. 1) was carried out within more-focused pH limits (pH 6 to 11). A total of 30 differentially expressed spots were found (white and blue circles and numbers). Molecular weight standards are indicated on the left, and pH values are indicated on the bottom.

It was apparent that the basic side of the 2-D gel contained a higher density of protein spots, which were not well resolved relative to other parts of the gel. A third experiment was therefore carried out using a more focused pH gradient (pH 6 to 11) to identify spots not detected using the broader pH range. An additional 30 protein spots were detected, of which 16 showed higher levels and 14 had decreased protein levels in A3HtrAOE in comparison to B31A3 (Fig. 2; Table S2). This experiment was carried out once.

10.1128/mBio.01136-18.2 TABLE S2 Identification of selected proteins from 2-D DIGE of B. burgdorferi 701 (pH 6 to 11). Download TABLE S2, DOCX file, 0.1 MB. Copyright © 2018 Coleman et al. 2018 Coleman et al. This content is distributed under the terms of the Creative Commons Attribution 4.0 International license.

A combined total of 36 protein spots that were reduced in A3HtrAOE and had protein level ratios above a 1.5-fold cutoff were chosen from both pH groups (Tables S1 and S2) for direct identification by means of mass spectrometry (MS; matrix-assisted laser desorption ionization–time of flight [MALDI-TOF]). We were specifically interested in these proteins as they could be direct substrates reduced through proteolysis. The significantly increased A3HtrAOE proteins, which may be the result of unknown interactions and are thus not the result of direct proteolysis by HtrABb, were not studied further (Tables 1 and 2).

We previously reported that standard 2-D electrophoresis analysis of B. burgdorferi B31A3 wild type and HtrA-overexpressing strain A3HtrAOE identified a likely HtrA substrate candidate with a pI, molecular mass, and MALDI-TOF mass spectrometry (MS) spectrum consistent with those of outer membrane protein P66 (20). In the current study, P66 resolved as four distinct apparently full-length proteins (spots 5 to 8) that ranged in pI from 5.8 to 6.25 (predicted, 5.90) (Fig. 1; Table 1). Several smaller P66 peptides were also observed (pI 6.4, 46 kDa, spot 82, and pI 5.8, 33 kDa, spot 83). Regardless of pI and size difference, all were reduced in A3HtrAOE (Fig. 1 and Tables 1 and S1). These results confirm, by different methodologies, that a rise in cellular HtrABb is associated with a decrease in P66 protein expressed by the HtrABb-overexpressing A3HtrAOE strain (20), thus establishing P66 as an important substrate for HtrABb. In the same previous study, the level of *p66* transcript in A3htrAOE was significantly reduced (3.3-fold, *P* ≤ 0.0001) in comparison to wild-type spirochetes. This result, in combination with the *in vitro* and *in vivo* degradation of P66 by HtrABb, suggested that this protease may be affecting the expression of P66 at the transcriptional and at the protein levels (20).

**TABLE 1:** Identification of selected proteins from 2-D DIGE of B. burgdorferi (pH 3 to 10)^a^

Spot no.	PER, expt 1/expt 2	Top-ranked protein (species)	Accession no.	Protein MW	Protein pI
5	−2.75/−3.77	Integral OMP p66 (B. burgdorferi B31)	gi|15594948	68,130	6.04
6	−3.02/−3.64	Membrane protein (B. burgdorferi)	gi|700323990	68,130	6.04
7	−2.94/−3.41	Membrane protein (B. burgdorferi)	gi|700323990	68,130	6.04
8	−1.45/−2.05	Integral OMP p66 (B. burgdorferi B31)	gi|15594948	68,130	6.04
9	−1.68/−2.06	ABC TSBP (B. burgdorferi)	gi|700323739	62,329	9.16
13	−1.55/1.27	Oligoendopeptidase F (B. burgdorferi)	gi|499192176	69,679	6.84
15	−1.23/−2.79	ABC TSBP (B. burgdorferi)	gi|700323739	62,329	9.16
16	−2.08/−1.95	Extracellular SBP (B. burgdorferi)	gi|482681275	62,319	9.12
19	−2.80/−3.07	Glycerol kinase	gi|6685462	55,571	7.02
20	−2.74/−3.53	Glycerol kinase	gi|6685462	55,571	7.02
21	−1.96/−2.33	Glycerol kinase	gi|6685462	55,571	7.02
24	−1.74/−2.42	Glycerol kinase	gi|6685462	55,571	7.02
25	−2.63/−3.08	GPDH (B. burgdorferi)	gi|480313620	54,478	8.89
26	−2.51/−2.89	GPDH (B. burgdorferi)	gi|480313658	55,223	8.88
38	−2.33/−1.31	GAPDH (B. burgdorferi)	gi|146743622	34,965	6.79
39	−2.17/−1.13	GAPDH	gi|3915702	36,232	7.74
46	−1.74/−1.77	Chain E, Lyme disease antigen OspA	gi|11514691	27,623	8.35
48	−1.83/−1.87	Putative lipoprotein (B. burgdorferi ZS7)	gi|218164577	36,439	8.88
51	−2.20/−2.56	Chain E, Lyme disease antigen OspA	gi|11514691	27,623	8.35
73	−1.99/−4.76	Peptide deformylase (B. burgdorferi)	gi|488738649	19,104	6.32
75	−1.53/−1.92	NDPK (B. burgdorferi)	gi|488620324	19,337	6.52
82	−1.83/−2.89	Integral OMP p66 (B. burgdorferi B31)	gi|15594948	68,130	6.04
83	−2.42/−3.27	Integral OMP p66 (B. burgdorferi B31)	gi|15594948	68,130	6.04
85	−2.43/−3.64	Integral OMP, partial (B. burgdorferi)	gi|697996210	26,233	6.04
86	−2.14/−2.13	Chain E, Lyme disease antigen OspA	gi|11514691	27,623	8.35
87	−2.81/−4.11	Antigen, p83/100 (B. burgdorferi Bol26)	gi|226233237	79,324	5.09
89	−1.99/−2.60	P41, partial (B. burgdorferi)	gi|483603	35,730	5.54

a Abbreviations: PER, protein expression ratio; OMP, outer membrane protein; TSBP, transporter substrate-binding protein; SBP, solute-binding protein; GPDH, glycerol 3-phosphate dehydrogenase; GAPDH, glyceraldehyde-3-phosphate dehydrogenase; NDPK, nucleoside-diphosphate kinase.

### HtrABb degrades DNA-binding protein Hbb.

The DNA-binding protein Hbb (BB0232) (31, 33) has been reported to interact with the *p66* promoter and may contribute to its regulation (32). Hbb, a basic transcription factor (pI 10.89; relative molecular weight [*M*_r_], 12,654) known to bind upstream of *p66* on the chromosome, was not detected in 2-D DIGE. The area where it is predicted to migrate in 2-D DIGE likely contains many proteins with similar molecular weights and isoelectric points, including histone-like proteins. The predicted proximity of the Hbb protein to the farthest cationic edge of the pH gradient further complicated its detection.

**FIG 3:**
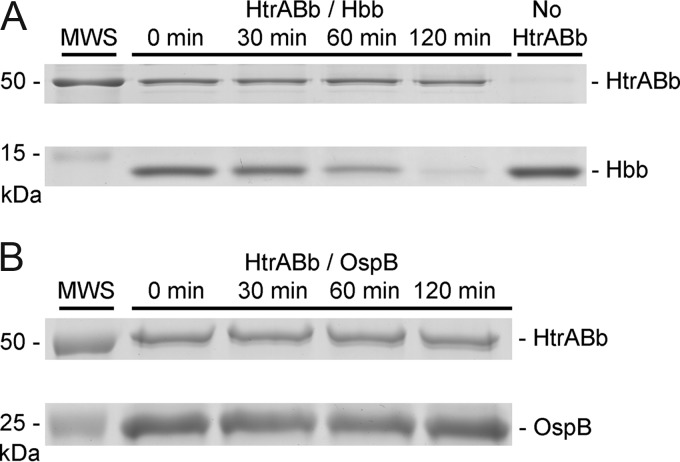
HtrABb degrades DNA-binding protein Hbb *in vitro*. (A) Purified recombinant Hbb (10 µg) was incubated at 37°C for 0, 30, 60, and 120 min with recombinant HtrABb (5 µg) in a 50-µl volume of DPBS. At each time point, 10-µl aliquots were boiled for 3 min with SDS-PAGE sample buffer to stop enzymatic activity. A negative-control digestion without HtrABb was incubated at the same time. Samples were run on a 15% SDS-PAGE gel and stained with Coomassie blue. (B) Purified recombinant outer surface protein B (OspB) was incubated with HtrABb as in panel A. Samples were run on a 12% SDS-PAGE gel and stained with Coomassie blue.

To determine whether HtrABb is involved in the regulation of P66, possibly through proteolytic regulation of Hbb, we conducted digestion experiments whereby recombinant HtrABb was coincubated with recombinant Hbb. In repeated experiments, HtrABb specifically degraded Hbb (Fig. 3A). HtrABb did not degrade recombinant OspB (Fig. 3B), demonstrating the substrate specificity of the protease. The degradation of Hbb, a putative transcription factor for P66 expression, represents another point of evidence indicating that HtrA regulates this protein through two pathways. This experiment was carried out two times with consistent results.

### The HtrABb-overexpressing strain has defective motility.

It was previously shown that a catalytically inactive form of HtrABb (HtrABb^S198A^) bound chemotaxis phosphatase CheX in immunoprecipitation assays (1). Subsequently, it was demonstrated that recombinant HtrABb degraded CheX *in vitro* (1). We therefore sought to confirm these results by 2-D DIGE. Attempts to show differential regulation of CheX in this manner were not successful as CheX (molecular weight [MW], 17,614) is an acidic protein (pI 4.15) that is outside the anionic range of the 2-D DIGE procedure.

**FIG 4:**
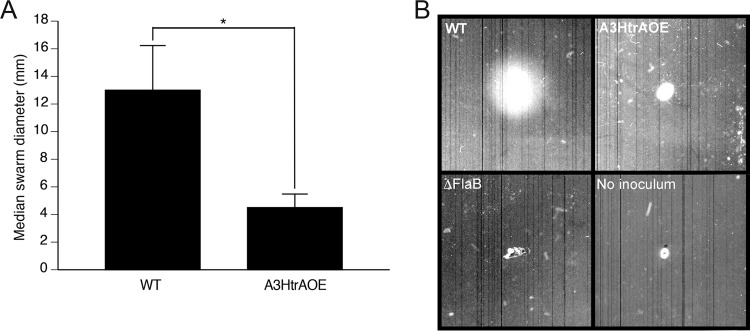
HtrA-overexpressing strain A3HtrAOE displayed a motility defect in comparison to WT B31A3, as demonstrated by plate swarm assay. The abilities of B. burgdorferi B31A3 and A3HtrAOE to swarm were analyzed in plate assays consisting of semisolid BSK medium diluted 1:10 in 0.35% agarose. (A) Following incubation (7 days, 35°C, 2.5% CO_2_), swarm diameters in individual plates for WT B31A3 (*n* = 12) and A3HtrAOE (*n* = 18) were measured and compared. Statistically relevant differences between the medians were determined by use of the Mann-Whitney test for nonparametric data where *P* is ≤0.0001. The error bars represent the standard deviations. (B) Images from representative experiments showing wild-type B31A3, A3HtrAOE, and Δ*flaB* strains as well as a no-inoculum control are shown.

Despite producing high levels of HtrA, the overexpressing strain exhibits normal motility when examined by dark-field microscopy. In addition, recovery of spirochetes from organ culture and quantitative PCR (qPCR) of mice at 21 days postinfection were indistinguishable from wild-type B31A3 (20). In an attempt to explain these findings further, we used motility swarm assays to investigate whether A3HtrAOE showed normal dissemination in culture dishes containing 10% Barbour-Stoenner-Kelly (BSK)–3.5% agarose semisolid medium (34). In repeated swarm assays (*n* = 15), wild-type B31A3 swarmed to a median diameter of 13 mm from the inoculation site. A3HtrAOE (*n* = 19), on the other hand, swarmed to a markedly reduced median diameter of 4.5 mm (*n* = 18). These results were statistically significant (*P* < 0.0001) (Fig. 4A and B) and represented a strong and unequivocal phenotype. The ΔFlaB strain was used as a nonmotile control (Fig. 4B).

**FIG 5:**
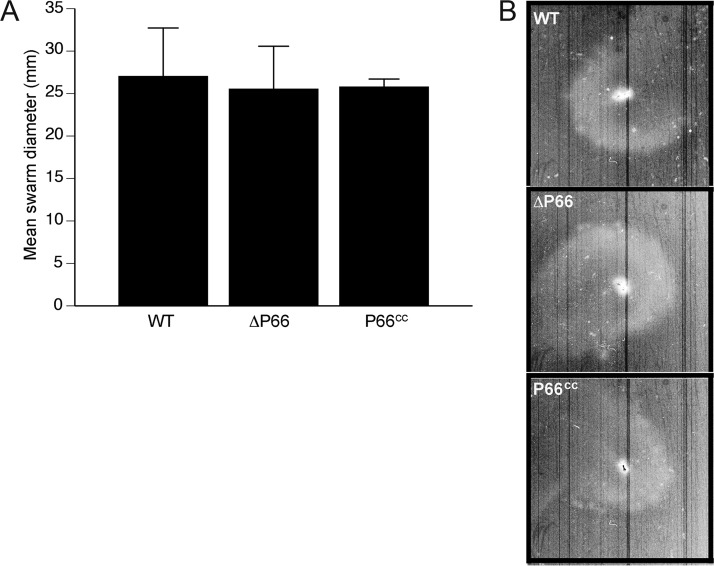
Loss of outer membrane protein P66 does not result in reduced motility. Swarm assays utilizing wild-type B31A3, P66 knockout strain (ΔP66), and the P66-complemented strain (P66^cc^) were carried out in an identical manner as those described in the Fig. 4 legend. (A) Following incubation (35°C, 2.5% CO_2_), swarm diameters in individual plates for WT B31A3 (*n* = 4), ΔP66 (*n* = 4), and P66^cc^ (*n* = 4) strains were measured and compared. The error bars represent the standard deviations. (B) Representative images are shown for WT, ΔP66, and P66^cc^ strains.

Given that A3HtrAOE exhibits a reduced level of P66, presumably as a consequence of expressing an increased level of HtrA, and is defective in motility, we investigated whether the loss of *p66* was the cause of the defect through the use of a motility swarm assay of a strain in which *p66* had been deleted (35). There was no difference in swarm diameter between wild-type B31A3 and the Δ*p66* strain (Fig. 5), thus eliminating a role for P66 in the B. burgdorferi swarm assay.

### 2-D difference gel electrophoresis reveals degradation of three glycolytic pathway enzymes in HtrABb-overexpressing strain A3HtrAOE.

In addition to confirming P66 as a substrate for HtrABb, 2-D DIGE of B31A3 wild type and A3HtrAOE identified a set of enzymes of the glycolytic pathway, consisting of glycerol kinase (GK) (spots 19 to 21 and 24), glycerol 3-phosphate dehydrogenase (GPDH) (spots 25 and 26), and glyceraldehyde-3-phosphate dehydrogenase (GAPDH) (spots 38 and 39) (Fig. 1 and 2; Tables 1 and 2) that were significantly reduced in A3HtrAOE compared to the B31A3 wild type. Diphosphate-fructose-6-phosphate 1-phosphotransferase (PFP, Table 2), an enzyme involved with carbohydrate metabolism that transfers phosphoryl groups reversibly to fructose, was also degraded in the 2-D DIGE.

**TABLE 2:** Identification of selected proteins from 2-D DIGE of B. burgdorferi (pH 6 to 11)^a^

Spot no.	PER	Top-ranked protein (species)	Accession no.	Protein MW	Protein pI
1	−1.54	Glycerol kinase	gi|6685462	55,571	7.02
3	−1.68	Membrane protein (B. burgdorferi)	gi|700323990	68,130	6.04
4	−1.55	ABC TSBP (B. burgdorferi)	gi|700323739	62,329	9.16
5	−1.51	PFP (B. burgdorferi 64b)	gi|223885464	62,393	6.17
6	−1.94	Glycerol kinase	gi|6685462	55,571	7.02
7	−2.49	Glycerol kinase	gi|6685462	55,571	7.02
9	−2.31	GAPDH (B. burgdorferi)	gi|146743616	35,206	7.25
16	−1.70	Chain E, Lyme disease antigen OspA	gi|11514691	27,623	8.35
17	−2.64	Chain E, Lyme disease antigen OspA	gi|11514691	27,623	8.35
18	−1.63	Chain E, Lyme disease antigen OspA	gi|11514691	27,623	8.35

a Abbreviations: PER, protein expression ratio; TSBP, transporter substrate-binding protein; PFP, diphosphate-fructose-6-phosphate 1-phosphotransferase; GAPDH, glyceraldehyde-3-phosphate dehydrogenase.

### A3HtrAOE has significantly reduced production of pyruvate.

**FIG 6:**
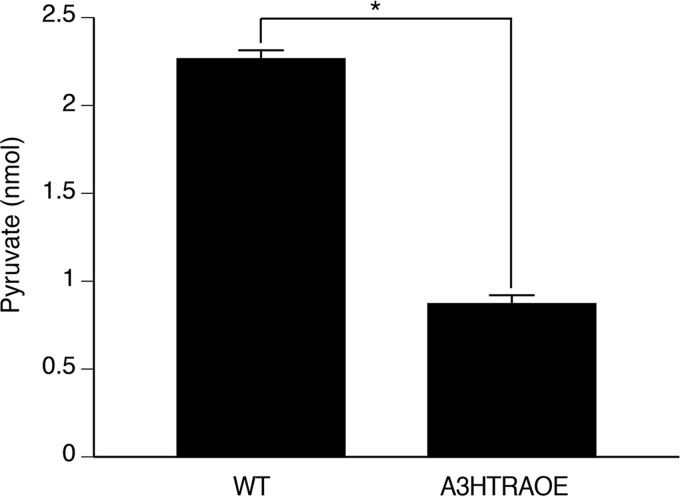
A3HtrAOE produces less pyruvate than wild-type B31A3. B. burgdorferi B31A3 and A3HtrAOE were analyzed by a standardized kit assay to determine if they produced equivalent levels of pyruvate during growth at 37°C. The data shown are a representative example of a total of three assays. The trend was the same for all three experiments. Statistically relevant differences between the means were determined by use of the two-tailed *t* test, where *P* was ≤0.0001 (*). The error bars represent the standard deviations.

In normal cells, the process of glycolysis results in a net positive output of pyruvate and ATP. To determine whether the observed impaired expression of GK, GPDH, and GAPDH in A3HtrAOE was reflected by a decreased output of glycolytic pathway end products, we tested and compared B31A3 wild type and A3HtrAOE for total pyruvate production. In mid-log-phase cultures, total production of cellular pyruvate was lower by 2.5-fold in A3HtrAOE than in B31A3 wild type, indicative of an impaired glycolytic pathway (Fig. 6). This experiment was carried out three times with consistent results.

## DISCUSSION

Our results confirm unequivocally that outer membrane protein P66 is an *in vivo* substrate for HtrABb as shown by 2-D DIGE. Moreover, the suggestion that P66 is also regulated at the transcriptional level was strengthened through the proteolysis of Hbb, a putative transcription factor for P66. From the foregoing, it is evident that HtrABb has a major role in the regulation of this outer membrane protein that has multiple functions in B. burgdorferi. Studies using differential culture conditions suggested that P66 production is regulated at the transcriptional as well as posttranscriptional level (29). This conclusion was also reached in our own study (20). P66 is one of the best-studied outer membrane proteins of B. burgdorferi. P66 is a surface-exposed protein and a ligand for β3-chain integrins (21–23). P66 also has porin activity (27, 28) by virtue of its outer membrane location and oligomeric composition (24, 26), featuring β-barrel structures (25, 26). The totality of this evidence strengthens the idea that P66 forms channels in the outer membrane, organized into oligomers of eight channels apiece (24).

**FIG 7:**
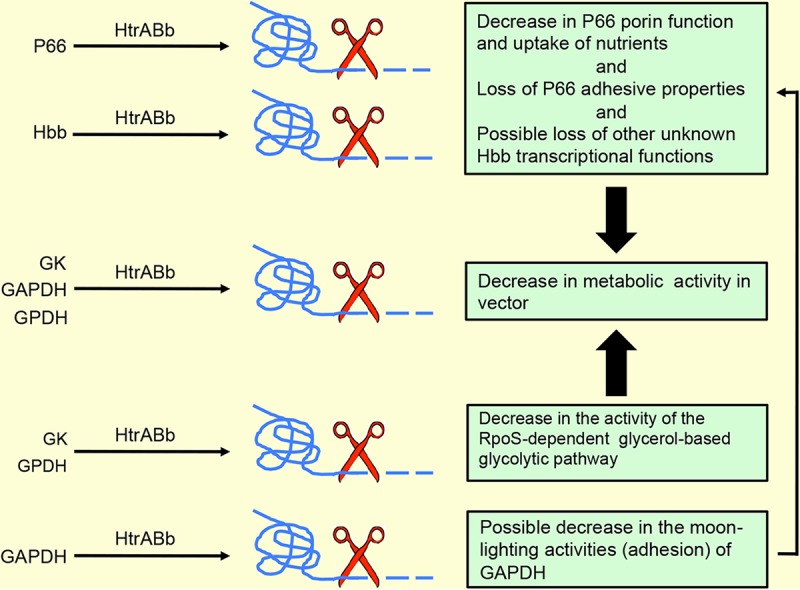
A model for the role of HtrABb through proteolysis of P66, Hbb, and three enzymes of the glycolytic pathway in a potential decrease in metabolic activity of B. burgdorferi. Proteolytic degradation of P66 could limit the amount of essential nutrients required for motility or for production of energy. Proteolytic degradation of glycolytic enzymes could result in a decrease of energy-producing molecules. The combined net effect of proteolysis of P66 and enzymes would be to prepare B. burgdorferi for the stage that requires lower metabolic activity.

P66 is not expressed while spirochetes are in the midgut of the unfed tick. Upon feeding with blood, P66 begins to be expressed, with production lasting until the blood meal has been digested (29). The changing expression of P66 in the life cycle of the spirochete may be regulated by HtrA, and a model is proposed for this function (Fig. 7).

Hbb is the homolog of DNA-binding proteins that include HU-like proteins and integration host factor or histone-like proteins (31). Proteins in this group are known to achieve transcriptional regulation by causing bends in DNA (33). Hbb of B. burgdorferi binds DNA as a homodimer with structural features that result in a large DNA bend. The *hbb* gene has been used for detection and phylogenetic analyses of B. burgdorferi (36, 37). Of direct importance to our study, recombinant Hbb bound the *p66* promoter region with high affinity and specificity in electrophoretic mobility shift assays. Using reverse transcription-PCR (RT-PCR) of *hbb* expression in tick midguts, a model supporting a repression role of *p66* by Hbb was advanced (32). Furthermore, the levels of Hbb are low, even in times of increased transcription during blood feeding in the tick, which is another reason for our inability to detect it in the 2-D DIGE. Our earlier results that transcription of *p66* was impaired (20) and the degradation of Hbb by HtrABb provide compelling evidence for a major role for this protease in the regulation of P66 expression. One of the roles of HtrABb is to shut down production of P66, and given that there is no expression of P66 in the unfed tick (29), it would appear that HtrABb contributes to the preparation of B. burgdorferi for its tick stage. Although not metabolically inert, B. burgdorferi in the tick midgut during the intermolt period has markedly reduced metabolic activity.

Our 2-D DIGE results provided several potential reasons for the swarm assay phenotype of A3HtrAOE. Degradation of P66 could have accounted for the lack of motility. In its role as a porin, P66 could transport essential nutrients that are required for motility, nutrients that would be lost if P66 was degraded. However, this was not the case, as the Δ*p66* strain displayed normal swarm assay motility. Degradation of CheX (phosphatase) is not likely to account for the swarm assay phenotype either, as lack of CheX would result in persisting motility.

For every molecule of glucose, the glycolytic pathway produces a net total of two molecules of pyruvate and two molecules of ATP. By use of 2-D DIGE, we showed that an overexpression of HtrABb results in reduced expression of three glycolytic pathway enzymes: GAPDH, glycerol 3-phosphate dehydrogenase, and glycerol kinase (GK). GAPDH, a direct participant in glycolysis, catalyzes the conversion of glyceraldehyde-3-phosphate (GAP) to 1,3-biphosphoglycerate in the sixth step of the glycolytic pathway. Before glycerol can enter the glycolytic pathway, it has to be converted into an intermediate product, dihydroxyacetone phosphate (DHAP). Glycerol kinase further catalyzes the transfer of a phosphate group, forming glycerol 3-phosphate. Glycerol 3-phosphate is dehydrogenated into DHAP through catalysis with glycerol 3-phosphate dehydrogenase and can enter the glycolytic pathway. Thus, all three enzymes degraded by HtrABb play a role in the eventual yield of pyruvate and a reduction in protein levels of any or all of the three enzymes conceivably would result in a corresponding reduction in the glycolytic output. In turn, degradation of these enzymes by HtrABb could have an important role in the swarm assay phenotype by limiting the amount of energy required by these spirochetes such as we showed with significantly reduced output of pyruvate in the A3HtrAOE. A model (Fig. 7) considers both the possible effects of the degradation of P66 and the enzymes of the glycolytic pathway by HtrABb in the swarm phenotype and in a possible decrease of metabolic activity. The above represents the more obvious explanations for the role of HtrABb in the degradation of the three glycolytic pathway enzymes, but these explanations need to be reconciled with the switch to an RpoS-dependent glycerol-based glycolysis pathway in ticks (38, 39), which utilizes the same enzymes used in the glycolysis of glucose. Utilization of the glycerol pathway in the tick would require glycerol kinase and glycerol 3-phosphate dehydrogenase, which were both degraded by HtrABb, so we are left with the unknown as to when in the life cycle of B. burgdorferi this degradation could actually occur. Modulation of glycolysis, in either a glucose-abundant (mammal) environment or a glucose-poor environment (tick midgut), could occur in the transitional stages of the life cycle such as entry into the tick or transmission into the mammal, where different levels of energy are required. However, at this point, we cannot provide any evidence for HtrABb-mediated proteolysis of enzymes of the glycolytic pathway *in vivo*, and the role of the protease in modulating glucose- or glycerol-based glycolysis will need to be studied further.

It is well known that some of the prokaryotic glycolytic enzymes exhibit more than one function, or moonlighting. Enzymes involved in metabolic regulation can localize to the bacterial surface and have additional biological properties in bacterial virulence (40). Moonlighting GAPDH is one such surface-located ligand for plasminogen (41). We presented evidence for possible moonlighting by the GAPDH of B. burgdorferi in addition to its traditional enzymatic role in the glycolytic pathway (42, 43). Thus, it is possible that HtrABb degrades only those enzymes that are extracytosolic. The latter option is known for other bacteria, notably Gram-positive bacteria, and this could occur in B. burgdorferi as well. There is a precedent for the degradation of glycolytic enzymes by HtrA. A mutation in the HtrA of Streptococcus mutans altered the surface expression of enolase and GAPDH by causing increased production of both enzymes, establishing them as the substrates of the protease (44).

Last, it is also possible that HtrABb does not interact directly with cytosolic enzymes at all but rather regulates unknown intermediate molecules that may be responsible for the changes observed.

The role of HtrABb in the modulation of the glycolytic pathway during the life cycle transitions of B. burgdorferi could be studied in depth with new metabolomics tools. Such studies would be enhanced with the use of a recently developed deletion mutant of HtrABb that had developmental and morphological defects at high temperatures and was not able to infect mice (45).

## MATERIALS AND METHODS

### Strains, media, and reagents.

The B31A3 strain of B. burgdorferi and B31A3-derived HtrABb-overexpressing strain A3HtrAOE (20) were used for all 2-D difference gel electrophoresis (2-D DIGE) experiments, as well as swarm motility and pyruvate assays. The P66 knockout strain (Δ*p66*) and Δ*p66*^cc^ complemented strain (*p66* reintroduced to its original locus on the chromosome) (35) were made in the B31A3 background. An FlaB knockout (flagellin B; Δ*flaB*) was used in swarm assays as a negative control (46). Recombinant Hbb protein (31, 47) was used along with HtrABb for proteolysis experiments. Recombinant OspB was used for enzymatic controls (48).

### 2-D DIGE of B. burgdorferi.

2-D DIGE and mass spectrometry protein identification were run by Applied Biomics (Hayward, CA).

### Isoelectric focusing and SDS-PAGE.

Spirochetes (5 × 10^9^) grown at 33°C (wild type [WT] and A3HtrAOE) were centrifuged and washed twice with phosphate-buffered saline (PBS). Our spirochetes are grown at this temperature in our laboratory. The pellets were resuspended in 100 µl of cell lysis buffer (30 mM Tris-HCl containing 7 M urea, 2 M thiourea, and 4% CHAPS {3-[(3-cholamidylpropyl)-dimethylammonio]-1-propanesulfonate}, pH 8.8) and sent to Applied Biomics (Hayward, CA) on dry ice for proteomic analysis. The samples were sonicated on ice, followed by shaking for 30 min at room temperature before centrifugation for 30 min at 4°C at 25,000 × *g* for collection of the supernatant (protein lysate). Lysate samples were diluted with the 2-D cell lysis buffer to a concentration of 6.0 mg/ml. Each lysate sample was covalently linked to a different cyanine fluor dye (green [Cy3] for B31A3 wild type and red [Cy5] for A3HtrAOE). For CyDye labeling, 30 µg of protein lysate was added to 1 µl of diluted CyDye (1:5 diluted with *N*,*N*-dimethylformamide [DMF], from 1-nmol/µl stock solution), vortexed, and kept on ice for 30 min in the dark. One microliter of 10 mM lysine was added, and the samples were incubated for another 15 min under the same conditions. Cy3- and Cy5-labeled samples were combined with an equal volume of 2× 2-D sample buffer (8 M urea, 4% CHAPS, 20 mg/ml dithiothreitol [DTT], 2% Pharmalytes, and trace amount of bromophenol blue), to which 100 µl of rehydration buffer (7 M urea, 2 M thiourea, 4% CHAPS, 20 mg/ml DTT, 1% Pharmalytes, and trace amount of bromophenol blue) was added to a final volume of 250 µl for the immobilized pH gradient strips (IPG strip, 13 cm; GE Healthcare Life Sciences, Chicago, IL).

The samples were mixed and separated according to pI using pH 3 to 10 or pH 6 to 11. Upon completion of the isoelectric focusing (IEF), the IPG strips were incubated in equilibration buffer 1 (50 mM Tris-HCl, containing 6 M urea, 30% glycerol, 2% SDS, trace amount of bromophenol blue, and 10 mg/ml dl-dithiothreitol at pH 8.8) for 15 min with slow shaking. The IPG strips were rinsed in equilibration buffer 2 (50 mM Tris-HCl containing 6 M urea, 30% glycerol, 2% SDS, trace amount of bromophenol blue, and 45 mg/ml iodoacetamide, at pH 8.8) for 10 min with gentle shaking. The second dimension was 12% SDS-PAGE. Running the differentially labeled control and experimental samples in the same IPG strip/gel eliminated intergel variation.

### Image scan and protein spot identification.

Image scans were done immediately following SDS-PAGE using a Typhoon Trio variable model imager system. The scanned images were analyzed by Image Quant TL software (GE Healthcare), and in-gel and cross-gel analyses were performed using DeCyder software version 6.5 (GE Healthcare). The ratio change of the protein level differential was obtained from in-gel DeCyder software analysis.

Protein spots were excised by the Ettan Spot Picker (GE Healthcare), and the gel spots were digested with modified porcine trypsin protease (Trypsin Gold; Promega, Fitchburg, WI). The resulting peptides were desalted with a Zip-Tip C_18_ column (Millipore), eluted with matrix solution (α-cyano-4-hydroxycinnamic acid, 5 mg/ml in 50% acetonitrile, 0.1% trifluoroacetic acid, 25 mM ammonium bicarbonate; 0.5 µl) and spotted on the MALDI plate. MALDI-TOF (MS) and TOF-TOF (tandem MS/MS) were performed on a 5800 mass spectrometer (AB Sciex, Framingham, MA). Both the resulting peptide mass and the associated fragmentation spectra were submitted to GPS Explorer version 3.5 equipped with the MASCOT search engine (Matrix Science, Boston, MA) to search the nonredundant database of the National Center for Biotechnology Information (NCBI nr) or the Swiss-Prot database. Searches were performed without constraining protein molecular weight or isoelectric point, with variable carbamidomethylation of cysteine and oxidation of methionine residues, and with one missed cleavage allowed in the search parameters. Confidence intervals greater than 95% were considered significant.

### Swarm plate assay for assessing spirochete motility.

Spirochete cell motility was determined by swarm plate assays as previously described (34), with some alterations. Semisolid swarm plates contained BSKII medium diluted to 1:10 in 0.35% autoclaved agarose LE in Dulbecco’s PBS (DPBS). Plates were prepared by the addition of 40 ml molten agarose-BSKII to 100- by 20-mm tissue culture dishes (Sarstedt, Newton, NC). Plates were allowed to solidify in a laminar flow hood. Early- to mid-log-phase BSKII cultures of B. burgdorferi (15 ml, 33°C) were harvested by centrifugation (4°C, 5,000 × *g*) and gently resuspended in fresh BSKII to a density of 1.5 × 10^9^/ml. Plates were inoculated with 7.5 × 10^6^ (5 µl) of spirochetes and incubated at 35°C and 2.5% CO_2_ for 7 days. Swarm diameter images were obtained by scanning in a Bio-Rad GS-800 calibrated densitometer, and swarm diameters were also measured. Wild type and A3HtrAOE as well as the ΔP66 strain and its complemented strain (P66^cc^) were used in the swarm assays. The ΔFlaB strain was used as a nonmotile control strain.

### Measurement of total cellular pyruvate.

Total cellular pyruvate was assayed using a pyruvate assay kit (Abcam, Cambridge, MA) in the colorimetric assay format. The purpose of the assay was to measure and compare the pyruvate outputs of B. burgdorferi B31A3 and A3HtrAOE. At the outset, the early- to mid-log-phase cultures grown in BSKII medium (this medium contains sodium pyruvate) were enumerated, and 4 × 10^10^ cells were washed with and resuspended in Dulbecco’s PBS followed by lysis in kit assay buffer. The protein concentration of each sample was then measured using the Pierce bicinchoninic acid (BCA) protein assay kit (Rockford, IL), and the results were used to further equilibrate the samples to ensure that the same amount of each sample was being assayed. The final number of B. burgdorferi bacteria per 96-well plate well (Greiner Bio-One, Monroe, NC) was 5 × 10^6^. The remainder of the procedure followed the kit instructions exactly. The plate was read in a VersaMax Tunable microplate reader (Molecular Devices, Sunnyvale, CA).

### HtrABb-mediated proteolysis of recombinant Hbb.

Digestion of recombinant Hbb protein was done as previously described for other proteins (20). Briefly, purified recombinant Hbb (10 µg) was incubated at 37°C for 0, 30, 60, and 120 min with purified recombinant HtrABb (5 µg) in a 50-µl volume of DPBS. At each time point, 10-µl aliquots were boiled for 3 min with SDS-PAGE sample buffer to stop enzymatic activity. A negative-control digestion without HtrABb was incubated at the same time. Samples were run on a 15% SDS-PAGE gel and stained with Coomassie blue. In addition, HtrABb, in order to demonstrate its substrate specificity, was incubated with purified recombinant outer surface protein B (which it does not degrade), and the samples were processed in the same manner as Hbb.
