# Supplementary material for: HtrA of Borrelia burgdorferi Leads to Decreased Swarm Motility and Decreased Production of Pyruvate
Source: mBio. 2018 Jul 10;9(4):e01136-18. doi: 10.1128/mBio.01136-18 (PMC6050954; doi:10.1128/mBio.01136-18)
Supplement: TABLE S1 [file mbo004183963st1.docx]

Table S1. 2-D DIGE Protein Expression Ratios (pH 3-10)^a^.

|  | **Protein Expression** | **Ratio^a^** |  |  | **Protein Expression** | **Ratio** |
| --- | --- | --- | --- | --- | --- | --- |
| **Spot No.** | **Expt. 1 (pH 3-10)** | **Expt. 2 (pH 3-10)** |  | **Spot No.** | **Expt. 1 (pH 3-10)** | **Expt. 2 (pH 3-10)** |
| 1 | 1.30 | 1.58 ^b^ |  | 51 | -2.20 | -2.56 |
| 2 | 1.22 | 1.31 |  | 52 | 1.12 | 1.16 |
| 3 | 1.62 | 1.37 |  | 53 | -1.11 | -1.99 |
| 4 | 1.42 | 1.67 |  | 54 | 1.09 | 1.00 |
| 5 | -2.75 | -3.77 |  | 55 | 2.34 | 1.22 |
| 6 | -3.02 | -3.64 |  | 56 | 4.63 | 3.09 |
| 7 | -2.94 | -3.41 |  | 57 | 1.63 | 2.39 |
| 8 | -1.45 | -2.05 |  | 58 | -1.26 | -1.31 |
| 9 | -1.68 | -2.06 |  | 59 | 2.05 | 1.88 |
| 10 | 1.43 | 1.39 |  | 60 | 1.94 | 1.79 |
| 11 | 1.12 | 1.13 |  | 61 | 2.25 | 2.34 |
| 12 | 1.12 | 1.20 |  | 62 | 2.11 | 1.67 |
| 13 | -1.55 | 1.27 |  | 63 | 1.33 | -1.10 |
| 14 | -1.21 | -1.22 |  | 64 | 1.06 | -1.10 |
| 15 | -1.23 | -2.79 |  | 65 | 2.71 | 1.51 |
| 16 | -2.08 | -1.95 |  | 66 | 4.02 | 4.57 |
| 17 | -1.23 | -1.22 |  | 67 | 1.34 | -1.43 |
| 18 | 1.12 | -1.47 |  | 68 | 2.42 | 3.02 |
| 19 | -2.80 | -3.07 |  | 69 | 1.95 | 2.30 |
| 20 | -2.74 | -3.53 |  | 70 | 3.12 | 1.18 |
| 21 | -1.96 | -2.33 |  | 71 | 4.07 | -1.54 |
| 22 | 1.50 | 1.51 |  | 72 | 3.15 | 4.95 |
| 23 | 1.14 | 1.23 |  | 73 | -1.99 | -4.76 |
| 24 | -1.74 | -2.42 |  | 74 | 1.31 | 1.63 |
| 25 | -2.63 | -3.08 |  | 75 | -1.53 | -1.92 |
| 26 | -2.51 | -2.89 |  | 76 | -1.34 | -1.40 |
| 27 | 1.15 | 1.45 |  | 77 | 2.18 | 2.49 |
| 28 | 1.15 | 1.20 |  | 78 | 2.05 | 1.74 |
| 29 | 1.34 | 1.07 |  | 79 | 1.24 | 1.33 |
| 30 | 1.73 | 1.14 |  | 80 | 1.06 | 1.18 |
| 31 | 1.59 | 1.75 |  | 81 | -1.39 | -1.85 |
| 32 | 1.77 | 1.54 |  | 82 | -1.83 | -2.89 |
| 33 | 1.83 | 1.00 |  | 83 | -2.42 | -3.27 |
| 34 | 1.11 | -1.07 |  | 84 | 1.81 | 1.78 |
| 35 | 1.29 | 1.08 |  | 85 | -2.43 | -3.64 |
| 36 | -1.08 | -1.07 |  | 86 | -2.14 | -2.13 |
| 37 | -1.23 | -1.16 |  | 87 | -2.81 | -4.11 |
| 38 | -2.33 | -1.31 |  | 88 | 1.63 | 1.78 |
| 39 | -2.17 | -1.13 |  | 89 | -1.99 | -2.60 |
| 40 | 2.20 | -1.01 |  | 90 | 1.49 | 2.18 |
| 41 | 2.08 | 2.39 |  | 91 | 1.62 | 2.09 |
| 42 | 1.46 | 1.05 |  | 92 | >1.5 | >1.5 |
| 43 | 2.95 | 2.62 |  |  |  |  |
| 44 | 1.95 | -1.06 |  |  |  |  |
| 45 | 1.21 | 1.45 |  |  |  |  |
| 46 | -1.74 | -1.77 |  |  |  |  |
| 47 | 1.05 | 1.10 |  |  |  |  |
| 48 | -1.83 | -1.87 |  |  |  |  |
| 49 | 1.97 | 1.10 |  |  |  |  |
| 50 | 1.10 | -1.01 |  |  |  |  |

^a^A3HtrAOE ÷ B31 wild type

^b^Red numerals indicate expression ratios above 1.5-fold cutoff

Negative sign indicates A3HtrAOE down-regulation relative to wild-type. A3HtrAOE up-regulation values relative to wild-type do not have a sign.
