# Supplementary material for: HtrA of Borrelia burgdorferi Leads to Decreased Swarm Motility and Decreased Production of Pyruvate
Source: mBio. 2018 Jul 10;9(4):e01136-18. doi: 10.1128/mBio.01136-18 (PMC6050954; doi:10.1128/mBio.01136-18)
Supplement: TABLE S2 [file mbo004183963st2.docx]

Table S2. 2-D DIGE Protein Expression Ratios (pH 6-11).

| **Spot No.** | **Protein Expression Ratio^a^**  **(pH 6-11)** |
| --- | --- |
| 1 | -1.54^b^ |
| 2 | -1.49 |
| 3 | -1.68 |
| 4 | -1.55 |
| 5 | -1.51 |
| 6 | -1.94 |
| 7 | -2.49 |
| 8 | 1.52 |
| 9 | -2.31 |
| 10 | 1.63 |
| 11 | 1.62 |
| 12 | 2.75 |
| 13 | 3.64 |
| 14 | 2.17 |
| 15 | 2.7 |
| 16 | -1.7 |
| 17 | -2.64 |
| 18 | -1.63 |
| 19 | -1.86 |
| 20 | -1.68 |
| 21 | 1.74 |
| 22 | 2.97 |
| 23 | 1.77 |
| 24 | 2.18 |
| 25 | 2.37 |
| 26 | 1.97 |
| 27 | 1.88 |
| 28 | -1.5 |
| 29 | 3.03 |
| 30 | 1.74 |

^a^A3HtrAOE ÷ B31 wild type

^b^Red numerals indicate expression ratios above 1.5-fold cutoff

Negative sign indicates A3HtrAOE down-regulation relative to wild-type. A3HtrAOE up-regulation values relative to wild-type do not have a sign.
